# Hypoxia-regulated exosomes mediate M2 macrophage polarization and promote metastasis in chondrosarcoma

**DOI:** 10.18632/aging.205230

**Published:** 2023-11-21

**Authors:** Sheng-Mou Hou, Chih-Yang Lin, Yi-Chin Fong, Chih-Hsin Tang

**Affiliations:** 1 Department of Research, Taiwan Blood Services Foundation, Taipei, Taiwan; 2 The Director’s Office, Shin Kong Wu Ho-Su Memorial Hospital, Taipei, Taiwan; 3 Translational Medicine Center, Shin-Kong Wu Ho-Su Memorial Hospital, Taipei, Taiwan; 4 Department of Sports Medicine, College of Health Care, China Medical University, Taichung, Taiwan; 5 Department of Orthopedic Surgery, China Medical University Beigang Hospital, Yunlin, Taiwan; 6 Department of Pharmacology, School of Medicine, China Medical University, Taichung, Taiwan; 7 Department of Biotechnology, College of Health Science, Asia University, Taichung, Taiwan; 8 Chinese Medicine Research Center, China Medical University, Taichung, Taiwan; 9 Department of Medical Research, China Medical University Hsinchu Hospital, Hsinchu, Taiwan

**Keywords:** chondrosarcoma, exosome, macrophage, metastasis

## Abstract

Chondrosarcoma is a primary malignant bone tumor. Traditional therapy is not very effective, and it is prone to metastasis in the late stage. The tumor microenvironment (TME) plays a key role in the progression and metastasis of chondrosarcoma, and hypoxia is one of the key factors in the formation of TME. However, the detailed mechanism of how hypoxia affects tumor progression and metastasis in chondrosarcoma is still not fully understood. In this study, we focused on the mechanism of interaction between hypoxic chondrosarcoma cells (SW1353) and macrophages. Our results suggest that hypoxia enhances the release of exosomes from chondrosarcoma cells. These hypoxia-induced exosomes promoted macrophage polarization towards the M2 phenotype, characterized by the expression of CD163 and CD206, but not the M1 phenotype, characterized by CD86 expression. Further analysis revealed that M2 macrophages polarized by exosomes expressed arginase-1 and feedback to chondrosarcoma cells to promote migration. These results suggest that chondrosarcoma cells secrete more exosomes in a hypoxic microenvironment, and these hypoxia-derived exosomes induce the polarization of macrophages into an M2 phenotype, ultimately promoting the metastatic behavior of chondrosarcoma cells.

## INTRODUCTION

Chondrosarcoma is a rare and malignant bone tumor that poses significant challenges in terms of prognosis and treatment. Conventional therapies like chemotherapy or radiotherapy have shown limited effectiveness in treating this type of cancer [1]. Ranked as the second most common primary bone malignancy, chondrosarcoma is recognized as one of the most challenging bone tumors to diagnose and treat. Typically, it predominantly affects adults between the ages of 40 and 60 and is commonly found near axial bones, such as the pelvis, ribs, and scapula. Additionally, it also frequently occurs at the proximal ends of the limbs, such as the proximal humerus (shoulder) and proximal femur (hip). Currently, surgical resection represents the mainstay treatment for chondrosarcoma, with survival rates varying depending on the tumor grade. In high-grade chondrosarcoma, there is an increased likelihood of distant metastasis and a consequently poorer prognosis. The reported 5-year survival rates for patients with grade 1, 2, or 3 conventional chondrosarcoma are 99%, 92%, and 77% respectively [2, 3]. No effective treatments exist for unresectable or metastatic chondrosarcoma, so there is a pressing need to develop a new targeted approach.

Metastasis, the spread of *in situ* tumors to distant sites or organs (e.g., lungs), is strongly influenced by the tumor microenvironment, particularly the presence of hypoxia and infiltrating inflammatory cells, such as macrophages [4, 5]. Hypoxia, characterized by low oxygen levels, is a common feature of solid tumors and plays a crucial role in the malignant progression of tumors. In the tumor microenvironment, when the oxygen pressure drops below 5–10 mmHg, it will cause hypoxic and promote cancer metastasis. Hypoxia-inducible factors (HIFs) are transcription factors that operate in the hypoxic environment of cells. It consists of two subunits, HIF-1α and HIF-2α, which play pivotal roles in the tumor microenvironment. HIF stimulates tumor angiogenesis by activating various signaling pathways to trigger the expression of vascular endothelial growth factor (VEGF). This process alleviates hypoxic conditions within the tumor and provides favorable conditions for tumor growth, metastasis, and angiogenesis [5, 6]. Tumor-associated macrophages (TAMs) are macrophages found in tumor tissue. TAMs play crucial roles in various aspects of tumor cell physiology and pathology, including tumor cell proliferation, invasion, metastasis, angiogenesis, immunosuppression, and drug resistance. The literature states that under hypoxic conditions, TAMs release a protein called macrophage migration inhibitory factor (MIF) [7]. MIF helps stabilize HIF-1α protein and promotes the degradation of the protective basement membrane around tumors. This degradation facilitates the escape of tumor cells from immune cells, enhancing their ability to metastasize to distant sites. Thus, TAMs contribute to the invasive behavior of cancer cells in the hypoxic tumor environment. However, the mechanism of chondrosarcoma metastasis and TAM has not been studied in detail, so inhibiting the expression of TAMs may be an important strategy to regulate the microenvironment of chondrosarcoma and prevent metastasis.

Macrophages are a type of immune-related stromal cells commonly found in and around tumors. They can exhibit different phenotypes and functions based on different signals from the microenvironment. It can be mainly divided into two types: M1 (classically activated macrophages) and M2 (alternately activated macrophages) [8]. Studies have shown that M1 macrophages are mainly involved in inflammation and immune responses, while M2 macrophages help in immunosuppression, tissue repair, and promotion of cancer progression [9–11]. In addition, M1 macrophages express inducible nitric oxide synthase (iNOS) and play a role in initiating immune responses, including the activation of T cells and natural killer (NK) cells [12]. In contrast, M2-type macrophages produce anti-inflammatory cytokines, such as interleukin (IL)-10 and upregulate the expression of arginase-1, which can promote tumor cell proliferation, metastasis, invasion and angiogenesis, inhibit immune response [13]. However, the exact mechanism by which chondrosarcoma regulates macrophage polarization and promotes their metastasis under hypoxic conditions is unknown. Therefore, studying the exact mechanism of chondrosarcoma and macrophage polarization may be a new direction for the treatment of chondrosarcoma metastasis. Our study provides evidence that exosomes secreted by chondrosarcoma cells can polarize macrophages under hypoxic conditions.

Exosomes are a type of extracellular vesicles primarily utilized as intercellular signal transmission mediators to regulate physiological and pathological mechanisms [14]. These vesicles consist of lipid bilayer membranes and carry various signaling factors (e.g., lipids, proteins, DNAs, messenger RNAs [mRNAs], and non-coding RNAs) that enable exomes to act as "messengers" between cells, facilitating local and systemic communication [15]. Moreover, tumor-derived exosomes can significantly impact tumor growth and invasion, local inflammation, and immune modulation [16, 17]. Hypoxic exosomes can promote the polarization of infiltrating myeloid cells into M2-like macrophages via a microRNA-mediated metabolic shift [18]. Our study therefore sought to determine whether hypoxia-induced tumor exosomes promote chondrosarcoma metastasis in the tumor microenvironment. We also aimed to elucidate the molecular mechanism that explains how M2 macrophages regulate the occurrence and development of chondrosarcoma, in order to provide a theoretical foundation for molecular-targeted therapy against this cancer.

## RESULTS

### Hypoxia increases the expression of chondrosarcoma cell-derived exosomes and induces macrophage M2 polarization

**Figure 1:**
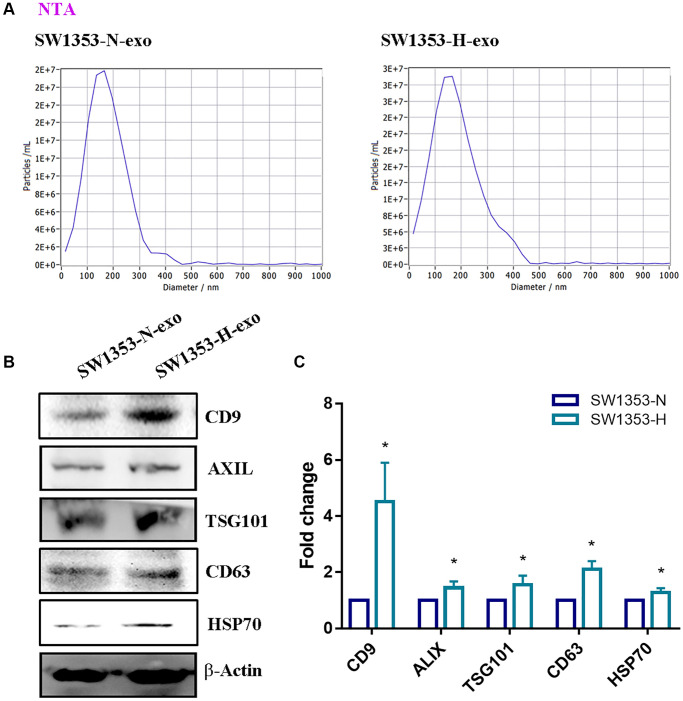
**Isolation of exosomes derived from chondrosarcoma cells.** (**A**) Identical concentrations of SW1353 cells were seeded under normoxic (O_2_ = 20%) and hypoxic (O_2_ = 1%) conditions for 48 h. Exosome (SW1353-N-exo and SW1353-H-exo) size was the characterization of exosomes by Nanoparticle Tracking Analysis (NTA). (**B**, **C**) Western blot analysis for exosomal proteins CD9, ALIX, TSG101, CD63, HSP70. All replicate number ≧ 3. ^*^p < 0.05 compared with the SW1353-N-exo group.

Our study aimed to investigate how hypoxia affects the secretion of exosomes from chondrosarcoma cancer cells and the ability of exosomes to induce macrophage M2 polarization. We seeded identical concentrations of SW1353 cells under both normoxic (SW1353-N) and hypoxic (SW1353-H) conditions and isolated exosomes from the conditioned media after 48 h. Nanoparticle tracking analysis (NTA) identified particles ranging from 30–150 nm in diameter (Figure 1A). The presence of exosomal proteins, including CD9, ALIX, TSG101, CD63, and HSP70, was validated through Western blot analysis of exosomal protein extracts (Figure 1B, 1C). Our findings indicate that hypoxia amplifies the release of exosomes from chondrosarcoma cells.

**Figure 2:**
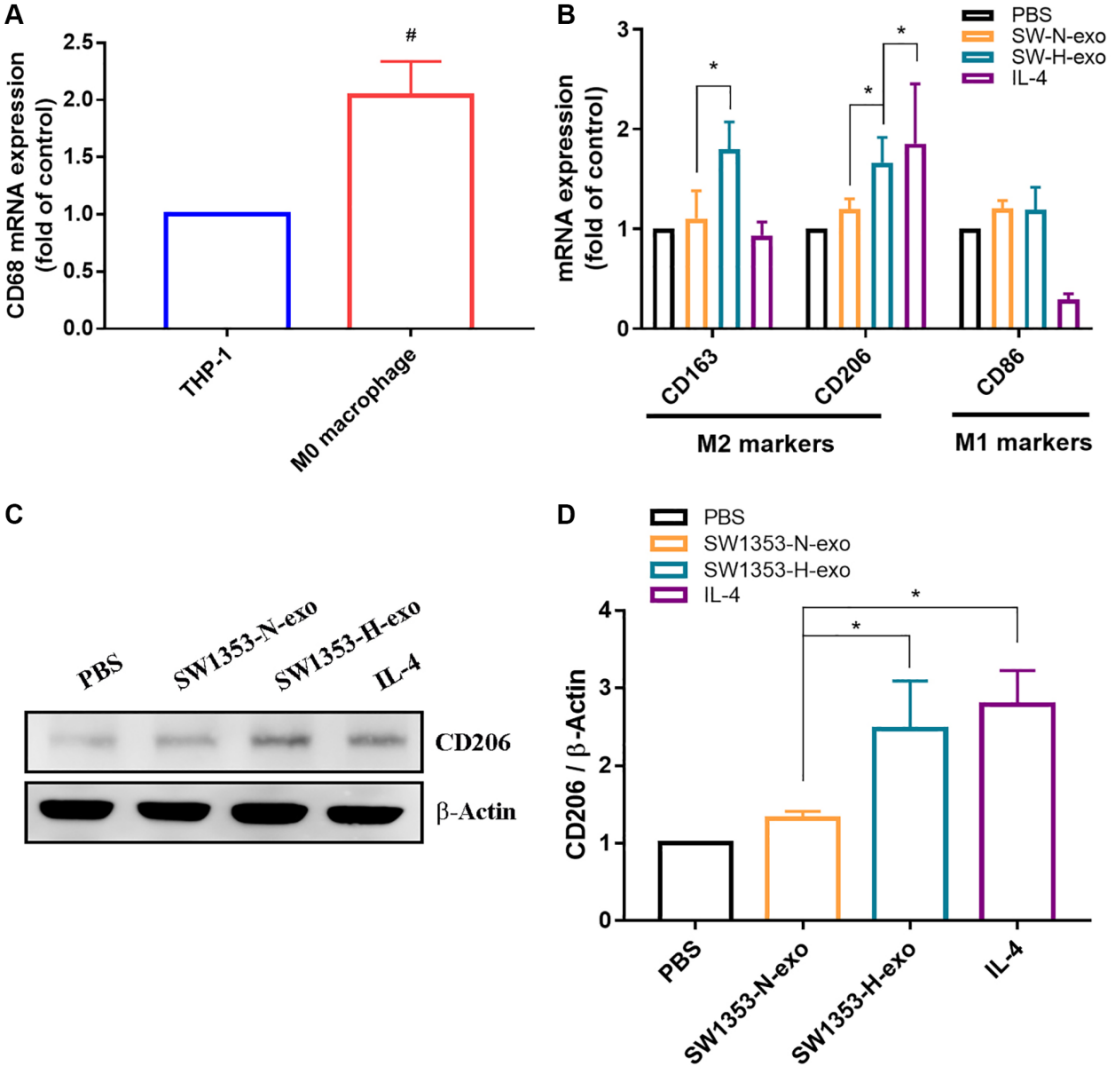
**Hypoxia promotes exosome secretion from chondrosarcoma cells that induce the polarization of M2 macrophages.** (**A**) qRT-PCR was used to detect the expression of CD68 (macrophage marker). (**B**) Macrophages were treated with SW1353-N-exo, SW1353-H-exo, or control (PBS and IL-4). After 48 h, qRT-PCR was applied using primers for M2 surface markers (CD163 and CD206) and an M1 surface marker (CD86). The group treated with IL-4 was used as a positive control. (**C**, **D**) Western blot analysis was used to detect the expression of M2 surface marker CD206. All replicate number ≧ 3. ^#^*p* < 0.05 compared with the THP-1 group; ^*^p < 0.05 compared with the SW1353-N-exo group.

**Figure 3:**
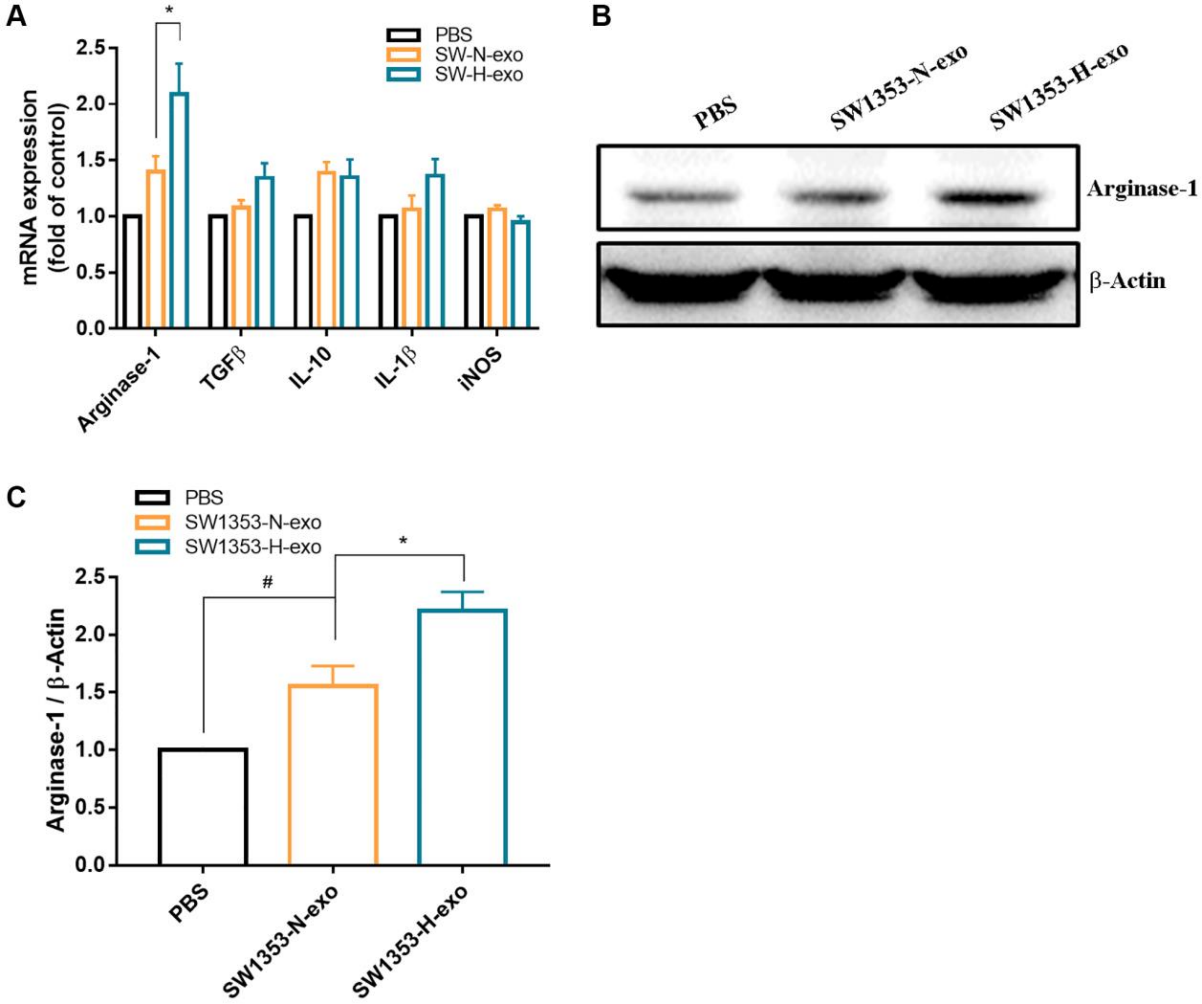
**Hypoxia promotes exosome secretion from chondrosarcoma cells that induce M2 macrophage expression arginase-1 protein.** (**A**) Macrophages were treated with SW1353-N-exo, SW1353-H-exo, or control (PBS). After 48 h, macrophage markers, including intracellular markers and cytokines (arginase-1, TGF-β, IL-10, IL-1β, iNOS), were detected by qRT-PCR. (**B**, **C**) Western blot analysis was used to detect the expression of the M2 intracellular marker arginase-1. All replicate number ≧ 3. ^#^*p* < 0.05 compared with the PBS group; ^*^p < 0.05 compared with the SW1353-N-exo group.

Hypoxia is a crucial characteristic of solid tumors, and secreted exosomes are known to deliver biological information to proximal or distal cells. Given that macrophages represent the predominant immune-related stromal cells within and surrounding tumors, our study aimed to explore the impact of hypoxic tumor-derived exosomes on macrophage polarization. To initiate this study, human THP-1 monocytes were treated with PMA (40 ng/mL) for 24 hours to induce macrophage differentiation, as shown in Figure 2A. Macrophages are characterized by their adherent morphology and expression of CD68. After confirming the differentiation of THP-1 into macrophages, we administered exosomes collected from SW1353 cells (under normal oxygen and hypoxia conditions) or performed PBS stimulation. The results showed that the expression levels of M2 surface markers including CD206 and CD163 induced by hypoxic exosome, IL-4 as positive controls, while the expression of the M1 surface marker (CD86) remained unchanged (Figure 2B–2D). Furthermore, in macrophages treated with SW1353-H-exo, levels of M2 intracellular marker (arginase-1) expression were increased, whereas no changes occurred in M1 surface marker (CD86) expression, M1 or M2 cytokine secretion (transforming growth factor beta [TGF-β], IL-10, IL-1β, iNOS) (Figure 3A–3C). Thus, hypoxia enhances exosome secretion from chondrosarcoma cells and activates macrophages towards the M2 phenotype.

### Hypoxic exosomes induce M2 macrophages to promote chondrosarcoma cell migration

**Figure 4:**
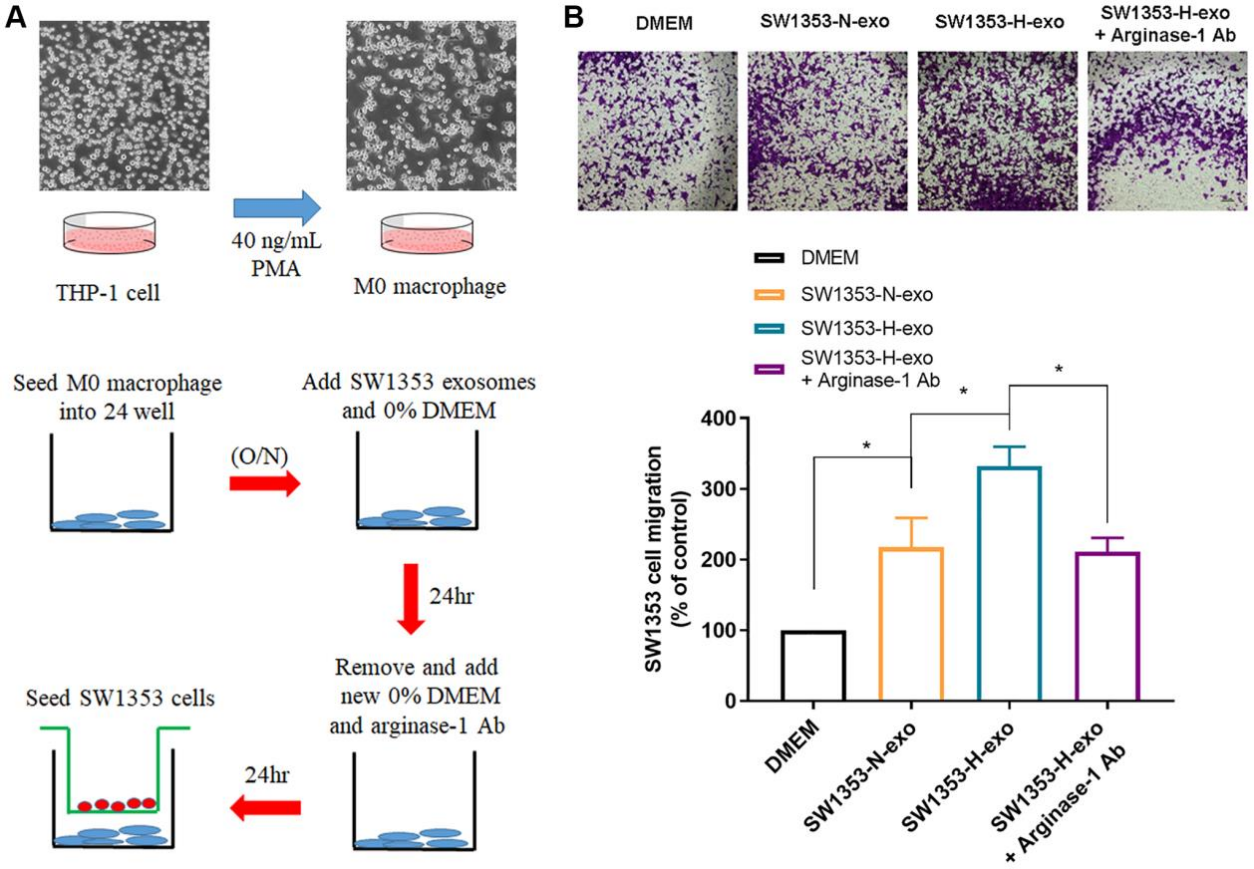
**M2 macrophages polarized by hypoxic exosomes promote the migration of chondrosarcoma cells.** (**A**) Schematic illustration of the *in vitro* indirect co-culture system. (**B**) The *in vitro* Transwell co-culture system determined the migratory capacity of chondrosarcoma cells (SW1353) co-cultured with exosome-treated macrophages. Representative photographs of migratory cells on the membrane are shown as morphometric analyses of migratory cells. All replicate number ≧ 3. ^#^*p* < 0.05 compared with the DMEM; ^*^p < 0.05 compared with the SW1353-N-exo group.

Subsequently, we proceeded to examine the influence of hypoxic chondrosarcoma cell-derived exosome-induced M2 macrophages on chondrosarcoma cell migration using an indirect co-culture system *in vitro* (Figure 4A). In the migratory Transwell assays, chondrosarcoma cell migration was significantly enhanced by M2 macrophages stimulated by hypoxic tumor-derived exosomes compared with normoxic exosomes (Figure 4B), pretreatment with M2 macrophages cell with Arginase-1 antibody significantly inhibited chondrosarcoma cell migration (Figure 4B). These findings suggest that hypoxia-induced exosomes activate M2 macrophages, which in turn promote chondrosarcoma cell migration.

## DISCUSSION

The prognosis for chondrosarcoma can vary greatly depending on the tumor subtype, with one research group reporting 5-year survival rates of 99%, 92%, and 77% for Grade 1, 2, and 3 conventional chondrosarcomas, respectively [3]. Histological assessment represents the definitive approach for grading chondrosarcoma and determining the suitable treatment following tumor resection. Nonetheless, this method’s reliability in diagnosis and prognosis may vary, highlighting the necessity for more objective and quantitative methodologies. Research is seeking biomarkers that can classify and grade chondrosarcoma, with the hope that such biomarkers will improve diagnostic reliability and better predict clinical behavior for therapeutic management [2]. Our study has demonstrated that hypoxic chondrosarcoma cell-derived exosomes induce macrophage M2 polarization and thereby promote chondrosarcoma cell migration. Understanding cancer cell-derived exosomal molecular mechanisms may lead to a novel therapeutic strategy for the treatment of chondrosarcoma metastasis.

Solid tumors are often characterized by hypoxia, which is linked to several cancer-related processes, such as angiogenesis, glycolysis, signaling, aggressive phenotypes, and poor prognosis [19–22]. Recent studies have established that exosomes released by cancer cells promote their malignant evolution, with hypoxia apparently stimulating the release of exosomes [17, 23]. For example, Zhang et al. reported that hypoxia induces the overexpression of circular RNA-101491 in exosomes derived from glioma cells. They proposed that the circ101491/miR-125b-5p/EDN1 regulatory axis might play a role in the malignant progression of glioma [24]. Additionally, it has been suggested that in a hypoxic microenvironment, nasopharyngeal carcinoma (NPC) cells release exosomal miR-455, which enhances vascular permeability and promotes metastasis by targeting zonula occluden-1 (ZO-1) [25]. Based on these discoveries, we hypothesized that hypoxia could augment the secretion of tumor exosomes and contribute to tumor progression. In this study, we demonstrate that hypoxia enhanced the secretion of exosomes from chondrosarcoma cells and promoted the malignant progression of chondrosarcoma cells. Thus, studying exosomal molecular mechanisms may serve as a novel marker and therapeutic target in chondrosarcoma progression. But it is worth noting that, although the market demand for exosome diagnostics and treatments has significantly increased, factors such as the cost of research, validation, and manufacturing, as well as future regulatory standards, may potentially impact future developments.

Tumor-derived exosomes have the ability to be internalized by macrophages within the tumor microenvironment, contributing to tumor progression and metastasis [26]. Tumor-derived exosomes have the ability to be internalized by macrophages within the tumor microenvironment, contributing to tumor progression and metastasis [26, 27]. Recently, several studies have reported that exosomes can modulate macrophage polarization, which in turn affects tumor metastasis. For instance, in colorectal cancer, exosomes derived from cells carry miR-934 and promote M2 polarization of macrophages, and thereby accelerate liver metastasis [28]. Exosomes obtained from hepatocellular carcinoma cells with hsa_circ_0074854 knockdown exhibit inhibitory effects on the M2 polarization of macrophages, thereby suppressing cancer cell migration and invasion [29]. Similarly, exosomes derived from ferroptosis-induced breast cancer cells impede M2 macrophage polarization, resulting in the suppression of breast cancer cell migration and invasion [30]. In our study, we observed that hypoxic exosomes stimulated the activation of macrophages towards the M2 phenotype, promoting chondrosarcoma cell migration. Our findings highlight the significance of hypoxic exosomes as a vital component of the tumor microenvironment and their involvement in mediating cellular cross-talk.

Hypoxia triggers unconventional protein secretion and the release of exosome-associated proteins in chondrosarcoma [2]. In addition, recent studies have found that exosomes derived from chondrosarcoma cells contain a long non-coding RNA called RAMP2-AS1 (lncRNA RAMP2-AS1), which mainly functions as a competitive endogenous RNA (ceRNA), the study confirmed that it can compete with miR-2355-5p. This ceRNA interaction positively regulates the proliferation, migration, and tube formation of human umbilical vein endothelial cells through the expression of vascular endothelial growth factor receptor 2 (VEGFR-2) [31]. However, our results confirmed that exosomes secreted by chondrosarcoma under hypoxic conditions can activate and promote the polarization of M2 macrophages, and M2 macrophages feedback to chondrosarcoma cells, resulting in increased migration of chondrosarcoma cells *in vitro*. Polarization of M2 macrophages leads to transcription of genes associated with the immunosuppressive macrophage phenotype in chondrosarcoma, including immunosuppressive markers such as arginase-1, TGF-β, and IL-10, as well as chemokines. Collectively, these factors may contribute to the progression and metastasis of chondrosarcoma.

Exosomes originating from chondrosarcomas serve as critical mediators in activating receptor cells and engaging in interactions with both nearby and distant cells, thereby facilitating the invasion and metastasis of chondrosarcoma. Therefore, utilizing these exosomes as a treatment approach for metastatic chondrosarcoma appears to be a reasonable strategy. Exosomes, due to their unique properties such as high biocompatibility, low toxicity, specificity, and stability, have the potential to be excellent carriers for treating metastatic chondrosarcoma [32]. One study identified miR-15a as the primary cargo of serum-derived exosomes, which can be internalized by osteosarcoma (OS) cells [33]. Additionally, elevated serum levels of RAMP2-AS1 were correlated with advanced stages and metastatic characteristics in chondrosarcoma patients, suggesting that RAMP2-AS1 emerges as a novel therapeutic target for chondrosarcoma within exosomes [23]. It is worth noting that some clinical trials have already achieved significant progress. For instance, research from the Musculoskeletal Tumor Center at Peking University People’s Hospital revealed that OS patients with lung metastasis exhibited higher programmed death-ligand 1 (PD-L1) and N-cadherin expression in serum exosomes compared to healthy subjects [34]. These studies indicate that exosomes may be potential targets for treating chondrosarcoma metastasis and represent one of the most promising approaches for clinical diagnosis and treatment.

**Figure 5:**
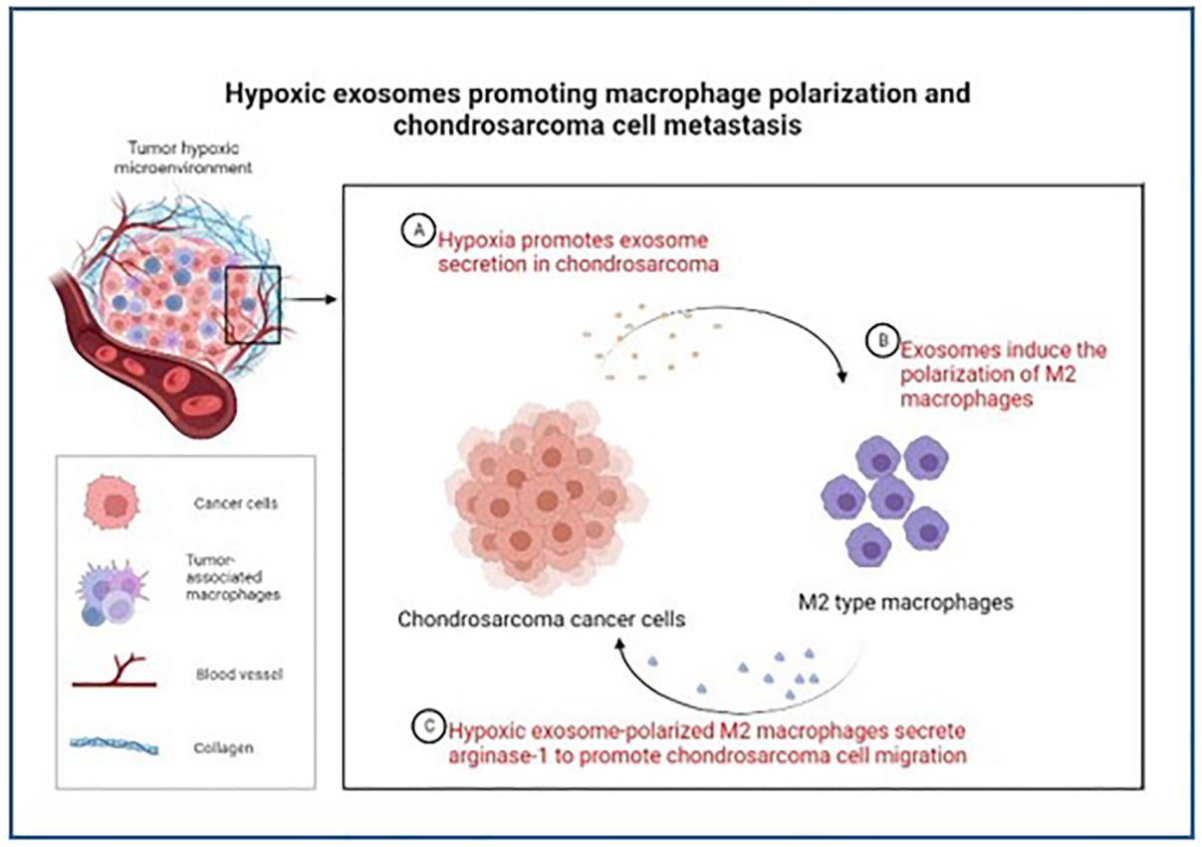
**Schematic model of hypoxic exosomes promoting macrophage polarization and chondrosarcoma cell metastasis.** Chondrosarcoma cell-derived hypoxic exosomes mediate macrophage M2 polarization and thereby facilitate the migration of chondrosarcoma cells.

In conclusion, our study demonstrates that exosomes released by hypoxic chondrosarcoma cells play an important role in polarizing macrophages to the M2 phenotype, and that polarized M2 macrophages express arginase-1 feedback to chondrosarcoma cells, ultimately promoting the migration of chondrosarcoma cells (Figure 5). These findings shed new light on the contribution of hypoxia-derived exosomes to the advancement of chondrosarcoma and highlight the potential of targeting exosomes as a therapeutic strategy for this disease. By interfering with the release or function of these exosomes, it may be possible to disrupt the M2 polarization of macrophages and impede the aggressive behavior of chondrosarcoma cells. Further research in this direction may offer valuable insights and potential therapeutic avenues for managing chondrosarcoma.

## MATERIALS AND METHODS

We obtained specific rabbit polyclonal antibodies for ALIX (Catalog No: A2215), TSG101 (Catalog No: A1692), and CD63 (Catalog No: A5274) from ABclonal (Woburn, MA, USA). The antibody for Arginase-1 (Catalog No: SC-166920) was purchased from Santa Cruz Biotechnology (Santa Cruz, CA, USA), while CD206 (Catalog No: 321102) antibody was obtained from BioLegend (San Diego, CA, USA). We ordered CD9 (Catalog No: NBP2-22187) and HSP70 antibodies (Catalog No: NB110-61582) from Novus Biologicals (Centennial, CO, USA) and β-actin antibody (Catalog No: a5441) from Sigma-Aldrich (St. Louis, MO, USA). The Pierce™ BCA Protein Assay Kit (Catalog No: 23225) was purchased from Thermo Scientific (Waltham, MA, USA). For cell culture experiments, we used Dulbecco’s modified Eagle’s medium (DMEM) and RPMI-1640 media, fetal bovine serum (FBS), and other cell culture reagents from Gibco-BRL Life Technologies (Grand Island, NY, USA). Recombinant human IL-4 (Catalog # 204-IL) was purchased from R&D Systems (Minneapolis, MN, USA). Phorbol 12-myristate 13-acetate (PMA; Catalog No: P1585), protease inhibitor (Catalog No: P8340), and all other chemicals were acquired from Sigma-Aldrich (St. Louis, MO, USA).

### Cell lines and culture

The SW1353 human chondrosarcoma cell line and the THP-1 human leukemia monocytes cell line were obtained from the American Type Culture Collection (ATCC) (Manassas, VA, USA). SW1353 cells were cultured in DMEM supplemented with 10% FBS. THP-1 cells were cultured in RPMI-1640 supplemented with 10% FBS. All media were supplemented with 100 U/ml penicillin and 100 μg/ml. All cells were maintained at 37°C in a humidified atmosphere with 5% CO_2_ [35, 36]. SW1353 cells were sub-cultured every 2–3 days by passing them when they reached 90-100% confluence using 0.05% trypsin.

To differentiate THP-1 monocytes into macrophages, they were incubated with phorbol 12-myristate 13-acetate (PMA) at a concentration of 40 ng/mL for 24 hours, followed by incubation in RPMI-1640 medium for another 24 hours. Macrophage M2 polarization was induced by treating the cells with interleukin-4 (IL-4) at a concentration of 20 ng/mL [37].

### Isolation of exosomes from cell culture supernatants

SW1353 cells were seeded at a density of 2 × 10^6^ cells per dish in 100 mm culture dishes. After reaching 90% confluence, the cells were incubated for 24 hours in a serum-free culture medium. Subsequently, under either hypoxic conditions (1% O_2_) or normal oxygen levels (20% O_2_), the serum culture medium was replaced and incubated for an additional 48 hours. Following the manufacturer’s instructions, extracellular vesicles were purified from the cell culture using the total exosome isolation reagent (from cell culture media) (Catalog No: 4478359) obtained from Life Technologies. The steps are as follows: Transfer the required volume of cell-free culture medium to a new tube and add 0.5 volumes of total extracellular vesicle isolation reagent. Incubate overnight at 2°C to 8°C. Centrifuge at 10,000 × g for 1 hour at 2°C to 8°C and remove the supernatant. Extracellular vesicles are contained in the pellet at the bottom of the tube (usually not visible). Resuspend the pellet in an appropriate volume of 1X PBS or a similar buffer and store at −80°C.

### Western blot analysis

To prepare the cellular lysates, we followed previously described protocols [38, 39]. In brief, the cells were lysed in RIPA buffer supplemented with a protease inhibitor cocktail. The protein concentrations were then determined using the BCA assay. Aliquots containing 30 μg of proteins were separated by sodium dodecyl sulfate-polyacrylamide gel electrophoresis (SDS-PAGE) and transferred onto polyvinylidene fluoride (PVDF) membrane filters. The PVDF membranes were blocked with Tris-Buffered Saline-Tween 20 (TBST) containing 4% non-fat milk at room temperature for 1 hour. Subsequently, the membranes were probed with primary antibodies against ALIX (1:1,000), Arginase-1 (1:2,000), CD206 (1:1,000), CD63 (1:1,000), CD9 (1:1,000), HSP70 (1:2,000), TSG101 (1:2,000), or β-actin (1:10,000) for 1 hour at room temperature, followed by three 5-minute washes with TBST. The membranes were then incubated with HRP-conjugated anti-rabbit or anti-mouse secondary antibodies (1:2,000) for 1 hour at room temperature. All Western blots within a panel are from the same experiment, and all blots were processed in parallel. Finally, the blot membranes were visualized using a Fujifilm LAS-3000 imaging system (Fujifilm, Tokyo, Japan) [40].

### Quantitative real-time polymerase chain reaction

Total RNA was extracted from SW1353 and THP-1 cells of equal passage number and density using TRIzolTM Reagent (MDBio Inc., Taipei, Taiwan). Subsequently, cDNA synthesis was carried out using the Invitrogen reverse transcription kit (Carlsbad, CA, USA) [41]. 2 μL of cDNA template, sequence-specific primers, and SYBR Green PCR Master Mix (Thermo Scientific, Waltham, MA, USA) were included in a total volume of 20 μL when analyzed using real-time PCR. Relative gene expression was calculated using a 2^−ΔΔCt^ method by normalization with glyceraldehyde 3-phosphate dehydrogenase (GAPDH) [42]. We used the PrimerBank to design qRT-PCR primers, and the primer sequences are listed in Table 1.

**Table 1:** Primer sequences for quantitative PCR (QPCR) used in this study.

**Genes (human)**	**Forward (5′-3′)**	**Reverse (5′-3′)**
Arginase-1	GTGGAAACTTGCATGGACAAC	AATCCTGGCACATCGGGAATC
CD68	GGAAATGCCACGGTTCATCCA	TGGGGTTCAGTACAGAGATGC
CD86	CTGCTCATCTATACACGGTTACC	GGAAACGTCGTACAGTTCTGTG
CD163	GCGGGAGAGTGGAAGTGAAAG	GTTACAAATCACAGAGACCGCT
CD206	GGGTTGCTATCACTCTCTATGC	TTTCTTGTCTGTTGCCGTAGTT
IL-10	GACTTTAAGGGTTACCTGGGTTG	TCACATGCGCCTTGATGTCTG
IL-1β	TTCGACACATGGGATAACGAGG	TTTTTGCTGTGAGTCCCGGAG
iNOS	TCATCCGCTATGCTGGCTAC	CCCGAAACCACTCGTATTTGG
TGF-β	CTAATGGTGGAAACCCACAACG	TATCGCCAGGAATTGTTGCTG

### Cell migration assay

To assess cell migration, we utilized 24-well Transwell culture plates (Costar, NY, USA) with a pore size of 8 μm, following the modified protocol based on the manufacturer’s instructions [43]. Initially, we seeded 1 × 10^4^ macrophages in the lower chamber of the Transwell plate, and subsequently added PBS along with exosomes secreted by chondrosarcoma cells. After 24 hours, the medium was replaced with fresh DMEM. Following that, we seeded 1.5 × 10^4^ chondrosarcoma cells into each well of the upper Transwell chamber. The cells were incubated for 18 hours at 37°C with 5% CO_2_ in a humidified incubator. Subsequently, the cells were fixed with a 3.7% formaldehyde solution at room temperature for 10 minutes, and stained with 0.05% crystal violet for an additional 10 minutes. Non-invasive cells on the top surface of the membrane were removed using a cotton swab. Cell migration was quantified by counting in three randomly selected microscopic views.

### Statistical analysis

The experiments were conducted either in duplicate or triplicate. The data are reported as the mean ± standard deviation (S.D.). Between-group differences were assessed using the Student’s *t*-test. A *p*-value of <0.05 was considered statistically significant.

### Data availability statement

The datasets utilized or analyzed during this study are accessible from the corresponding authors upon reasonable request.
